# “Hang the Flesh off the Bones”: Cultivating an “Ideal Body” in Taijiquan and Neigong

**DOI:** 10.3390/ijerph18094417

**Published:** 2021-04-21

**Authors:** Xiujie Ma, George Jennings

**Affiliations:** 1 Chinese Guoshu Academy, Chengdu Sport University, Chengdu 610041, China; ma.xiujie@outlook.com; 2 School of Wushu, Chengdu Sport University, Chengdu 610041, China; 3 Cardiff School of Sport and Health Sciences, Cardiff Metropolitan University, Cardiff CF23 6XD, Wales, UK

**Keywords:** mind-body, re-education, physical education, pedagogy, practice

## Abstract

In a globalized, media-driven society, people are being exposed to different cultural and philosophical ideas. In Europe, the School of Internal Arts (pseudonym) follows key principles of the ancient Chinese text The Yijinjing (The Muscle-Tendon Change Classic) “Skeleton up, flesh down”, in its online and offline pedagogy. This article draws on an ongoing ethnographic, netnographic and cross-cultural investigation of the transmission of knowledge in this atypical association that combines Taijiquan with a range of practices such as Qigong, body loosening exercises and meditation. Exploring the ideal body cultivated by the students, we describe and illustrate key (and often overlooked) body areas—namely the spine, scapula, Kua and feet, which are continually worked on in the School of Internal Arts’ exercise-based pedagogy. We argue that Neigong and Taijiquan, rather than being forms of physical education, are vehicles for adult physical re-education. This re-education offers space in which mind-body tension built over the life course are systematically released through specific forms of attentive, meditative exercise to lay the foundations for a strong, powerful body for martial artistry and health.

## 1. Bodies of Contrast in Taijiquan

“Hang the flesh off the bones” is a striking mantra that Author 2 (George) first encountered in October 2016, when he checked a potential fieldwork site for ethnographic information on the martial, meditative and healing art of Taijiquan (Tai Chi Chuan). This began to form the basis of an interdisciplinary, social scientific and empirical, qualitative project examining (a) how adult British students (re)learn about their anatomy through Taijiquan and (b) how another group learns about the anatomy of weaponry (such as medieval long swords) through historical European martial arts (HEMA). Over the last two years, Author 2 has immersed himself in the weekly classes and social events of the two case study schools, and this particular article deals with the case of Taijiquan in an effort to uncover some of the fundamental transformative exercises used to develop the idealized body in this art.

Since the official start of the project in spring 2019, the mantra of “hang the flesh off the bones” and its variants have continued to be uttered in a slow and soothing manner by the amiable and gentle instructor, David (pseudonym), who runs twice-weekly classes and regular workshops on Taijiquan, and related “internal” health and spiritual arts such as Neigong, in the United Kingdom. The unusual command of “hang the flesh off the bones” literally asks the student to follow “a sequence of events” by first using their intention (Yi) and awareness (Ting) to pull up their own skeleton from the crown point (and the occipital bone) of the cranium while eventually dropping the sacrum and tailbone, and then to imagine relaxing their musculature with gravity in order to sink the mass into the ground—a key principle for success in this art, known as Song. Whereas the former action of Ting is possible via setting and maintaining correct posture, the latter, relaxed control of Song is a quality that must develop over months or even years. 

The utterance “hang the flesh off the bones” is part of a series of unusual commands that form part of David’s inclusive yet strict pedagogy to develop the Taijiquan body—a soft, relaxed, yet alert bodymind. This bodymind is one in which the mind and body have been unified through a process of self-cultivation to develop the person in a holistic manner [1] as understood through different models of the mind-body connection, such as the energy systems seen in East Asian pedagogies [2]. This sequence of events forms the logic behind David’s pedagogy involving a step-by-step process of transforming his students in a holistic and long-term manner.

As Ryan notes [3], Taijiquan in Britain is commonly associated with health and wellbeing rather than martial artistry and self-defense. Within the health, fitness and wellness industries, there is an array of martial activities available for the discerning student, and “the School of Inner Arts” (pseudonym) is one of many Western interpretations of Taijiquan as taught as a strict, systematic awareness. The School’s activities differ from the more popular, simplified versions of the art developed in the 1950s for state-controlled health in the People’s Republic of China (PRC), and could be regarded as a reinvention of tradition. Indeed, Bowman [4], drawing on Ryan [3] and Frank’s [5] analysis, has recently reflected that Taijiquan is a quintessential example of the paradigm of a “traditional Asian martial art” that is actually a tradition of invention. One such invention is the combination of Taijiquan with other practices not normally seen elsewhere, such as meditation and specific approaches to self-cultivation. 

As Frank [5] has noted in his ethnographic research in China and the USA, Taijiquan is commonly connected to notions of “the little old Chinese man” and medicine, where old Asian bodies, moving slowly to heal themselves, are seen as the archetype of the martial art. This is undoubtedly the consequence of public perception of slightly built, elderly Chinese teachers depicted in Smith’s [6] early text, including his master, Zheng Manqing, who developed his own version of the Yang family short form via his interpretation of the art and its “internal” principles. Conversely, yet connected to this lineage, the School of Internal Arts is led by Malcolm Reeves (pseudonym), a charismatic, athletic, white British man and accomplished Taijiquan practitioner in his late 30s who has studied the martial arts since early childhood. Through his travels to China and South East Asia, Malcolm became a disciple of several reputed teachers of the Yang family style, and now teaches in Portugal and Sweden, where he leads his reputed international organization while writing numerous books and translations of Chinese treatises or ‘classics’ such as the Daodejing. He also hosts a regular podcast and YouTube series with thousands of followers and subscribers around the world—many of them having relocated to train with him full-time. As such, Malcolm represents a new generation of Western “influencers” in Taijiquan, spirituality and wellness. 

In Malcolm’s particular ‘internal’ martial arts organization concerned with a Daoist cosmology of wellness, ecology and spirituality, the ideal body is not one of overt muscular contraction and outer strength, but one of expansion and inner power in which the visible contours of the body slowly disappear (and reappear). Contrasting with the Western logic of medicine and bodily training, this body is supposedly a powerful body, and through being powerful, is a healthy body. The Taijiquan body, according to Malcolm’s studies and public interpretations of the Chinese medical theory of the Yijinjing (“the Muscle-Tendon Change Classic”), as explained on his YouTube channel, will eventually develop a tendon-like quality in which the practitioner develops strength, mobility and explosiveness alongside many mental qualities of a relaxed alertness. As an animal lover, Malcolm’s senior student and representative, David, likens this mind-body quality to a cat waiting to spring on a mouse: it is relaxed and completely focused on its task, never distracted and ready to pounce when necessary. 

It is important to note that the art now known as Taijiquan has developed since the 17th century in China under various names and formats. The slow, supple movement of modern Taijiquan is deeply rooted in traditional Chinese cultural thought, philosophy, medicine and martial arts [7]. The form of Taijiquan practiced today may be understood as traditional Chinese martial arts-based calisthenics, supplemented with a range of related exercises and physical practices (stretching, self-massage and Qigong), which can be regarded as a constitution and integration of traditional Chinese philosophy and health principles through breathing techniques and martial arts [8]. Within China and across the Chinese diaspora, Taijiquan has long been regarded as a Chinese “cultural treasure”, and this adds to a growing global interest in the martial arts as a form of living cultural heritage now recognized by UNESCO [9]. The Chinese philosophy drawn on in Taijiquan is mainly based on Confucianism, Daoism and Zhouyi, which takes the Taiji and Yin-Yang dialectical concepts in Daoist philosophy as the core ideas. The central doctrine of Daoism is focused on mental tranquility, and its goal is to achieve longevity by meditation and lifestyle modification [10] (pp. 342–346). 

With its competing histories, modalities and philosophies, Taijiquan includes different family styles (Chen, Yang, Wu武, Wu吴, Sun, He) and combinations and deviations from these. Taijiquan is currently thought to have developed from a military training exercise into a Daoist-informed art for a multitude of purposes, such as the training of actors [11]. As one of G’s former informants in another influential organization [12] once put it, Taijiquan “is a broad church” [13]: from the practical approach focusing on self-defense applications to the more accessible health-oriented art. The School of Internal Arts sits somewhere along the middle of this spectrum; it follows the Yang family tradition of Yang Chengfu (Zheng Manqing/Huang Shan Sheng lineage) for the 37-posture short form and the lesser known Yang Shaohuo for the grueling and more martial long form. Our analysis of pedagogy and practice in this article focuses on the former, more accessible training in public classes, as David does not have permission from his teacher to openly teach the long form. 

The ideal Taijiquan body permits great variety in terms of size, shape and outer appearance, as it is chiefly concerned with internal feel and function of movement. The eventually rotund body of the mature Yang Chengfu led him to develop a “large frame” for these techniques, but his physique would be regarded as morbidly obese in today’s society. Other late masters, such as the slightly-built Zheng Manqing (in his later years), conformed to the stereotype of what Adam Frank [5] explored in terms of age and ethnicity: the seemingly frail, little, old Chinese man who actually has a powerful body. It is important to note, however, that each martial art has its own idealized body. The physique of mixed martial arts (MMA) fighters is typically toned and callused—capable of giving and receiving pain [14]. The Aikido body, in contrast, is one that is ideally bottom-heavy and dense, making it difficult for the practitioner to be thrown due to a low center of gravity [15]. These ideal bodies are informed by specific philosophies of the body, health and life in general. In the next section, we examine one key text on bodily transformation, the Yijinjing, that has been reinterpreted by Malcom Reeves in order to guide all the training undertaken in the School. 

This article draws on ongoing ethnographic fieldwork on the School of Internal Arts to highlight several key exercises used to develop specific elements of the idealized Taijiquan body. These help address our leading question: how, as adults, are Westerners learning to re-educate their varied, individual bodies through prolonged and intense Taijiquan and Neigong training? As a collaboration between a Chinese and British martial arts researcher, the paper first outlines the research on Yijinjing and Taijiquan in China before moving to account for the ethnographic methods and our cross-cultural, multilingual analysis. We then offer a detailed description and illustration of exercises for the spine, scapula, Kua (胯) and feet which form the logical basis of particular body conditioning in the art of Taijiquan and the School of Internal Arts’ corresponding approach to Neigong: what we call the Taijiquan-Neigong body. We then offer some conclusions and future directions for research on the re-education of the body through practices and pedagogies informed by Eastern philosophy.

## 2. Taijiquan-Neigong Pedagogy and the Yijinjing 

As with Taijiquan, there are different opinions on the origin of the Yijinjing. From the current Chinese-language literature, most scholars believe it was created by the mystical Indian monk, Bodhidharma. According to legend, Bodhidharma came to China in 526 AD and eventually arrived at the Songshan Shaolin Temple. In order to alleviate the stasis of energy and blood caused by Zen sitting meditation practice, he supposedly practiced martial arts and Dao Yin techniques to move the muscles and bones, and modified and improved them, forming a fitness method named “Yijinjing”. Secretly spread among the Shaolin monks [10], the purported original purpose of Yijinjing was, thus, to reduce the fatigue and sleepiness caused by Zen practice to a certain extent, so the created movements are mainly joint extension exercises such as stretching and kicking. “Yi” means change, “Jin” refers to meridians and muscles and bones and “Jing” means ways and methods. In terms of its etymology, Yijinjing is thus a method to change the muscles and bones (and thereby the practitioner). In the process of the spread of the traditional Yijinjing, it has been studied from different perspectives such as religion, Yin-Yang and the five elements, and different schools have been created [16].

In the 1980s, “Qigong fever” emerged in China, and Qigong exercises were unprecedentedly popular, involving all parts of the country. In order to ensure the healthy and orderly development of Qigong fitness activities, and to make them scientific, standardized and legal, in 1996, the National Sports Committee of China first proposed the concepts of “social qigong”, “health qigong”, and “qigong medical treatment”. In 2000, the State Sports General Administration again coined the term “Health Qigong”, a traditionalist national body culture that combines physical activity, breathing and psychological adjustment as the main forms of exercise. This is a component of the long-standing Chinese culture. In June 2001, the Health Qigong Management Center of the State General Administration of Sports was established, and the Yijinjing was adapted into the new exercise method “Health Qigong-Yijinjing”. This is one of the first four sets of Health Qigong exercises promoted by the Chinese national government [17].

Nevertheless, Chinese scholars rarely conduct research on Yijinjing and Taijiquan outside China [18,19], such as on the spread and development of these practices and the meanings around the fitness effect of Yijinjing and Taijiquan on foreigners. This is accompanied by a lack of research into the local integration and development of Yijinjing and Taijiquan. Chinese scholars generally use the literature methods and experimental methods to study Yijinjing and Taijiquan [20,21,22,23,24,25,26], but eschew, for example, the type of ethnographic research used in our article.

Taijiquan has started to be used in Western countries such as Britain as an adapted exercise in prisons [27] or as a morning routine in primary schools [28]. This is a relatively new and non-standardized phenomenon, and it is not something that the cohort of mainly white British students in the School of Internal Arts learned as children in their formal physical education lessons as part of the UK National Curriculum [29]. Instead, their education in Taijiquan began as mature adults, aged from their early thirties to mid-sixties, after earlier exposure to Qigong and martial arts such as Karate or Aikido. Their bodies, minds and selves are molded into the School’s specific ideal based on the eclectic approach to Yang family Taijiquan and Neigong—the dispositions expected of the institutional martial habitus that adds to each person’s biographies [30]. 

Malcolm is now turning his attention to digital broadcasts explaining what he regards as the true interpretation of the more medical text on transformation, the Yijinjing. For Malcolm and his followers, the modern understanding of the (continually edited and illustrated) Yijinjing is incorrect, as for him, the original text does not prescribe a set of exercises, but is instead an introduction to a distinct set of principles. Following this logic, by adhering to the principles of the Yijinjing, a Taijiquan practitioner is seen to be practicing in the correct, internal manner. According to this interpretation of his own beloved mantra, “skeleton up, flesh down”, the body’s tissues will lengthen, and become more elastic and stronger through the hanging of the flesh over the bones. Following this logic, the physique of the practitioner will eventually possess “a tendon-like quality” that is powerful and explosive: like David’s analogy of the cat waiting for a mouse.

This article works from the premise that physical education does not stop upon the last day of school, as it can be argued to be a lifelong pursuit, like education in general. Moreover, just as there can be physical education, we see the potential for physical re-education of the human being in later, alternative forms of pedagogy such as Taijiquan. As such, as part of a special international, interdisciplinary and intercultural issue of the *International Journal of Environmental Research and Public Health* (IJERPH) on physical education, this article explores a form of re-education about the body, health and movement in the martial and meditative art of Taijiquan. It specifically focuses on David’s evening classes and online recorded classes during the lockdown and local COVID-19 restrictions in the UK. Through thematic analysis, we argue that a particular approach to Taijiquan as an experiential and folkloric body culture [31] shapes new understandings of seemingly rudimentary activities such as standing still, walking and moving with poise and control. Taking an eclectic theoretical approach from an interdisciplinary lens, we draw on two years of ethnographic field notes as well as interviews with David and video analysis from Malcolm’s podcasts in order to examine his alternative pedagogy, philosophy and the phrases that unite the Daoist worldview with the stillness and movement in this martial art. In the next section, we outline the research project and how the linguistic pedagogy was studied. We then turn to the analysis of the phrases linked to specific practices and techniques of the body, designed to develop particular aspects of the Taijiquan-Neigong body. This leads to a discussion, not just of what a body can become through the use of such concepts as linguistic bodies [32], but, as Spatz [33] questions, what a body can do. In this case, with a soft, relaxed, alert and powerful body, an exponent can move in an explosive fashion for self-defense. 

## 3. The Cross-Cultural, Collaborative Study

As well as being an extension of Author 2 (George)’s dual ethnographic study of HEMA and Taijiquan, this project is part of a wider collaboration between Chinese and British martial arts scholars seeking to uncover the ways in which Chinese Taijiquan culture is being transmitted in the British context. More specifically, this article follows the principles of traditional ethnography, which is, quite literally, the in-depth study and written representation of a group of people with a common culture. As set out in Hammersely and Atkinson, ethnographic fieldwork involves immersing oneself into the social life of a group of people to study their interactions, organization and practices via a combination of participation, observation, interviews and other qualitative data collection techniques [34]. This rich qualitative data is analysed on a regular basis in order to determine themes for further observations and note taking in the fieldwork site. The majority of the rich, descriptive data comes from field notes written during and directly after the social and embodied interactions with the research participants—all of which is intended to make the strange familiar (in an anthropological sense) and the familiar strange (in a sociological sense) [35], as we will explain.

Ethnography is a key research strategy in the expanding field of martial arts studies, and has taken a keen interest in the body and embodiment for many decades [36]. As a linguistic ethnography, its emerging research question is around the theme of language—namely, how Chinese linguistic terms and concepts are embodied and lived by Western practitioners (hailing from Britain, Eastern Europe, the USA, Spain and South Africa). As such, it explores ideas around human beings as linguistic bodies [35] that draw on concepts from different languages and embody these in their practice. Due to the gradual development of the bodymind in Taijiquan through its slow, systematic pedagogy, this project was planned for five years according to clear yet overlapping stages of: (1) fieldwork; (2) personal autophenomenographic reflections; (3) one-on-one interviews; (4) focus groups, and (5) online media analysis and documentary analysis. Because of the emergent nature of ethnography as an immersive and seemingly “messy” tradition [37], these stages have been altered to blend online and offline ethnography as seen in this article [38]. This follows continued immersion in a culture and intersubjective interactions in the social world online, which is now known as netnography [39].

The setting of the School of Internal Arts was selected after George settled into a new city, and where the institutions was recommended as a place where Taijiquan was taken very seriously in a traditionalist fashion. It was a convenience sample by chance, as G could walk to the classes and stay after the training sessions for friendly discussions with his fellow students. This coincided with his simultaneous ethnographic study of historical European martial arts (HEMA), which enabled a comparison of two very different kinds of martial arts, institutions, teachers and students—a blend of the relatively familiar (Taijiquan) and the strange (HEMA) for George, who is an experienced martial artist with a background in the Asian styles such as Wing Chun, several styles of Taijiquan, Taekwondo, Kendo and Mexican Xilam. However, the approach taken to Taijiquan in David’s class was unfamiliar to George, who was not accustomed to the focus on the scapula and the Kua (see Discussion). After several months of initial learning in the School, the ethnography officially began in March 2019 through the direct participant observation of George, a white able-bodied British man in his mid-thirties who joined David’s popular class as a beginner in this particular form of Taijiquan and a complete newcomer to the concept of Neigong. Taking the position of an apprentice, like many other ethnographers of mind-body practices [40], George gained the trust of the group and built rapport with David as the main gatekeeper of this branch of the international network. In addition to the twice-weekly evening classes held in a primary school and refugee support center, George engaged in regular meetings with his teacher, and is now undertaking one-on-one interviews with the core members of the school. 

From March 2020, with the advent of the COVID-19 pandemic and lockdowns in the UK, George’s fieldwork turned online by adopting a netnographic strategy of taking part in the online classes (through Zoom) and analyzing segments of the class deliberately recorded and shared with the students. George has undertaken regular thematic analysis of the field notes be re-familiarizing himself with the material and finding overlapping and recurring themes, such as embodied learning, transformation/change, Daoist cosmology and the role of humor in the pedagogy. This led him to work with Author 1 (Xiujie), a Chinese martial arts researcher and native Chinese speaker.

During his research visit to Britain, Xiujie joined one of the classes as an observer and participant, which enabled us to discuss the content and delivery of the Taijiquan and Neigong classes. He is a Chinese man in his mid-thirties who has practiced various forms of Chinese martial arts for more than 20 years. Xiujie also participated in local Taijiquan classes in the UK, which provided some data support for the ethnographic research and comparative thematic and linguistic analysis. Later, in order to make the research progress smoothly, George and Xiujie regularly communicated and negotiated the research process through online discussions over Skype, which facilitated the discussion of embodied technique and embodied knowledge [41]. This was helpful for George’s ethnographic analysis, as he was becoming very familiar with the approach taken by David; meanwhile, for Xiujie, the perspective taken on Taijiquan and the Yijinjing was radically different and strange, despite him being a native Chinese speaker. These online, cross-continental interactions enabled a cross-cultural dialogue on the different ideas around the Taijiquan and Neigong body, including Chinese/Daoist anatomy, the energetic or subtle body and the role of related practices surrounding Taijiquan. Such terms are nearly impossible to express purely in words, which is one limitation of the present form of representation as a modified realist tale [42] showing our analysis and involvement in the research process. This can be seen by our reflexive journal extract below, which illustrates the range of analogies that David employs to convey complex ideas, which in turn are expressed in a physical fashion:

*Using David’s analogy verbalized in class, George took out a wine glass and poured water into it. He said that David believed that if the wine glass is the external form of Taiji, then water is the inner essence of Taiji. Water can be poured into wine glasses, teacups, kettles, or bowls. But the inner essence of water has not changed, and only the outer manifestation of the load has changed. In other words, no matter the external appearance (routine) of the Chen family, Yang family, Wu style, Wu family and Sun family and their differences, the inner essence of Taijiquan is the same. Therefore, David believes that the focus of attention should be how to teach the true inner essence of Taijiquan to students, rather than excessive pursuit of external forms of expression*.

(Field notes, 28 November 2020)

The visual aid of the analogy in action helps the reader understand the idea that external forms are vehicles for the development of Taijiquan. In the following discussion, we turn to the interconnected teachings of Malcolm and his older apprentice David in order to examine: (1) the theory behind the School’s pedagogical practices and (2) how specific body parts are re-developed and reconceptualized through Chinese-language concepts as part of the process of re-education. In the second section, as with the analogy above, we have used visual aids through several photographs depicting key snapshots of several of the fundamental exercises described in each subsection. 

## 4. Discussion: Transforming the Students’ Bodies into a Taijiquan-Neigong Body

### 4.1. The Philosophy behind the School of Internal Arts

*“Taijiquan or Tai Chi is primarily known for its slow graceful movements and relaxing health benefits. However, Taijiquan is much more than this and works on a number of levels. It is a martial art that uses relaxed power to overcome a greater force and also a path in which to develop our consciousness. When we study Taijiquan along the classical Daoist threefold path, we bring together health, martial and consciousness practices to attain deep and meaningful change in our lives*.

*Nei Gong, which translates as ‘internal development’ in Chinese, is a process of change that works with the physical, energetic and consciousness bodies, to balance our nature and give us good health. A number of moving and static exercises are used in the process to clear and awaken the energy system; these include, Qi Gong, Dao Yins and breathing exercises. We aim to awaken our energy system via the lower Dan Tien, which sits below the navel.”*

This quotation comes from an official flyer promoting the School of Internal Arts’ two main activities: Taijiquan and Neigong. David handed the flyer to George when they met for one of their breakfast discussions on the meanings behind the two arts. The form of traditionalist or “authentic” Yang Taijiquan that is proudly promoted by the institution does not focus on the overtly martial version of the system, not does it provide a very accessible, easy version of movement for beginners. Instead, the instructors teach the art in what they see as the middle of the spectrum of the aforementioned broad church: in a disciplined manner that builds on basic movement exercises designed to prepare the body and mind (bodymind) for Taijiquan as understood through Malcolm’s translations of key Chinese principles such as Song (“the systematic release of tension”) and Ting (“the conscious awareness that goes into everything you do”). 

As Bowman [4] has noted, many Asian-language terms for techniques and concepts are not readily translated across countries. He offers the example of a Polish Judoka who felt uncomfortable with the English approach to pronouncing Japanese techniques. In Chinese martial arts, “Ting” (often known as Ting Jin in mainland China) is well known in Cantonese arts such as Wing Chun as a form of “listening energy”. Accompanying the principle of Ting is its lesser-known concept of “Song” which is used in order to systematically relax and lower the body’s center of gravity. The muscles, meridians and bones of the whole body are thereby stretched and lengthened. The various parts of the body are held in the best structure and are relatively long and relatively stable. By stretching and releasing, flexible elasticity is obtained. “Song” is not a state of laziness and laxity, nor a state of softness. Real relaxation means that the mind (意), sense (气) and strength (力) sink into the soles of the feet, and when they go deep into the ground the practitioner should feel that something rises from the soles of the feet, gradually filling the whole body, and pulling them up. Only with the feeling of leading upwards will the experience of sinking the whole body downwards be deepened. Only then can practitioners truly understand the essentials of Taijiquan, which is “rooted in the feet, sent through the legs, dominated at the waist, and shaped in the fingers” (其根在脚, 发于腿, 主宰于腰, 形于手指) [43] (p. 13). Taijiquan’s Kung Fu (skill achieved through time and effort) or “the marriage to the pursuit of skill” in Malcolm’s terms can also be said to be “Song” Kung Fu. Indeed, David often compares to Taijiquan to boxing: “A boxer’s basic skill is the punch. They learn to punch all day, whereas in Taijiquan, we learn to sink the mass”. In other words, a Western boxer’s Kung Fu lies in their ability to punch instinctively while the Taijiquan practitioner is concerned with sinking their mass so as to be hard to move in combat.

According to modern and popular (mis)conceptions, “Chinese boxing” or Chinese martial arts are generally divided into two rather divisive categories: “Internal Boxing” and “External Boxing” [44] (p. 249). The history and politics behind this division has been debated [45], but these ideas remain in contemporary Chinese martial arts circles, with practitioners often referring to their previous “external martial arts” training. Taijiquan is based on the theory of Taiji, while Baguazhang is based on the theory of Eight Diagrams, and Xingyiquan is based on the theory of Five Elements, which means that they all belong to the social category of “Inner Boxing” [46]. Therefore, it can be said that Neigong is the main training content of internal boxing, while Taijiquan is a type of internal boxing that expresses a distinct, idealized Taijiquan-Neigong body.

Bound by the principles of Song and Ting, the School of Internal Arts syllabus is vast, and can take over a decade or more to complete, yet alone master. From two years’ fieldwork to date, George has undergone a great variety of exercises connecting to Taijiquan and other arts such as Baguazhang—some of which appear for a matter of weeks, only to reappear a year later. However, the most commonly honed body parts and exercises are: (1) the spinal column via the spinal wave exercise; (2) the scapula and palms through the palm push and polishing glass exercises; (3) the Kua via Kua work and weight transfer/the eight gates, and (4) the feet (and sinking of the mass) via standing postures. We explain and discuss each of these essential foundational exercises in turn by focusing on parts of the body as in Bates’ [47] study of people living with, moving with and managing chronic illness. This linguistic body, as expressed by an emphasis on the spoken word, is explained in terms of the linguistic and philosophical principles inspired by the Yijinjing. We begin with the spinal wave, a non-martial preparatory exercise that can be practiced alone, at any time and place, like many other elements of this body pedagogy. These are described in detail, following the ethnographic tradition, while utilizing some images of George in action for illustrative purposes.

### 4.2. Opening the Vertebrae: The Spinal Wave

Contemporary Britain is a very sedentary society in which sitting constitutes a large part of most people’s day, and “bad backs” or lower back pain are the cause of a significant period of time off work. Even the National Health Service (NHS) have called for a reduction in the time spent sitting in order to reduce the high risk of serious illness [48]. This sedentary lifestyle and also over-specialization in movement (as in many sports) leads to compressed vertebra—something that David seeks to remedy in his classes. Committed students come from different martial arts backgrounds, such as Wing Chun, Aikido and lesser-known styles such as Chen Hsin—all of which have shaped the body in some way. For George, the rapid, forward movements of Wing Chun coupled with weight training in his adolescence had led his body to be quite toned yet slightly hunched, thereby limiting his mobility. Seen through a Daoist lens, the Taijiquan body is seen from inside, focusing on the links between organs and wellbeing, and this begins with work on the spinal column in order to develop good posture and elasticity around the body, which later enables something understood as “inner power”. 

**Figure 1:**
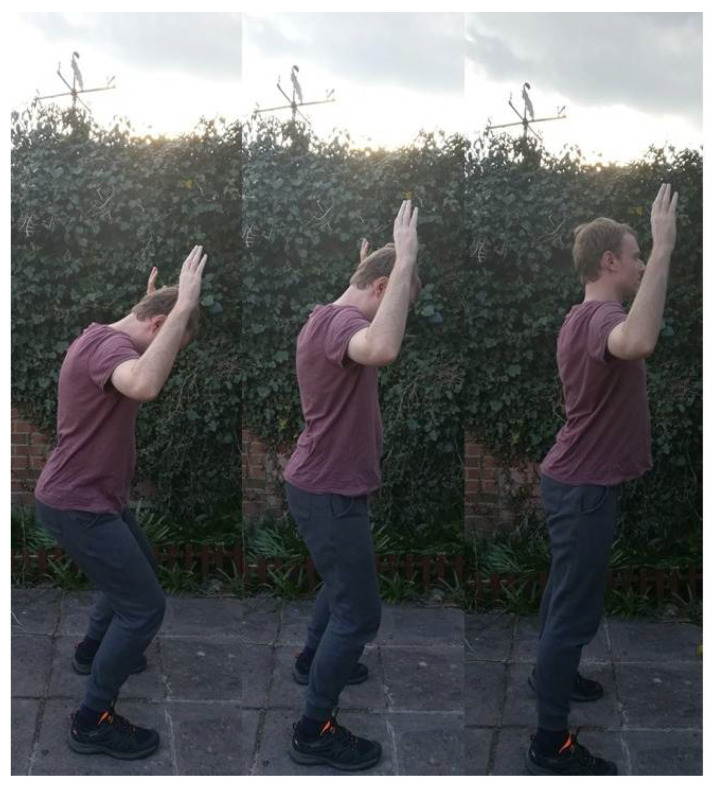
The Spinal Wave Sequence.

Figure 1 depicts the “spinal wave”, which is a gentle, rhythmic movement that initiates in the coccyx (or “tailbone” as it is referred to in class) and moves up the sacrum, lumbar and other regions of the spine until it reaches the occipital bone at the base of the skull. Some students might also envisage being raised at the crown point at the top of the skull “by a golden thread”, or being pulled up by a hook on the occipital bone at the back of the skull. In a typical evening class, David works in front of his 12–15 Taijiquan students lined up in two rows who are working in unison from a seemingly hunched, lifeless position, as in a puppet without a puppeteer pulling on the strings. Once their body is fully erect by stretching their spine upwards from the coccyx to the skull, they slump back down by folding the head forwards and sinking the tailbone down to bend the knees slightly. This initiates the process once again. Students then repeat the action dozens of times, while David adds variety by asking them to raise their hands above their heads while completing the motion. In Figure 1, George has deliberately selected this version of the exercise to emphasize the elongation of the spine from the profile position in three snapshots. On some occasions, student perform the exercise at a 45-degree angle. One might hear clicks in between the vertebra as they start to open. Indeed, David often shares a sense of wonder about this process, with comments such as: “Open up the space between the vertebrae”, reiterating the fact that “it’s so important to create space between the vertebrae”. Indeed, in one online class, David stressed that “there’s no reason for you to shrink as you get older”, as this can be prevented by softening the bunched up areas of the spinal column through these exercises. Hence Taijiquan exercises, such as those seen in this discussion, are designed to create more space within the inner body.

Unlike many postures in the syllabus (such as the all-important Wuji posture in the Neigong classes), this spinal wave is not given a Chinese term, but is nonetheless a cornerstone exercise for the class. It is normally used as part of the warmup after the basic limbering exercises, but it is also used to ease the body after prolonged periods of standing and form sequences. George has used this exercise not only in class but in between bouts of writing and marking, as an academic who uses his laptop for extensive periods of time while seated. He has heard clicks in different places across the spinal column (which are higher when the hands are raised), which have moved location throughout his two years as a student of David. Indeed, George deliberately worked on the exercise in between writing extracts of this discussion after analyzing his field notes on how David explained the importance of the movement in his usual soothing tone. George has also started the spinal wave before the beginning of a class (at 6:55, before it started at 7 p.m.), as students typically arrive early to ready their bodies for the intense training. “Spinal wave…let’s do the spinal wave, George!” said Rich, an excitable, confident, intermediate student in his fifties who moved to stand by George’s right side one evening, emulating the movement with a beaming smile. Rich made the exercise a shared one, as in the regular class, rather than George’s solo routine of warming up. 

### 4.3. Mobilising the Scapula: The Palm Stretch and Polishing Glass

From the spine comes another vital element of the Taijiquan-Neigong body: the scapula or “the true shoulder” as David calls it. David spends considerable time in class working on spreading and dropping the scapula in order for his students to become more mobile. Indeed, one of David’s students once joked to him: “Scapula and Kua, scapula and Kua, those are your two favorite words. You mention them all the time!” As they learn new postures, students are therefore encouraged to focus their attention on the spine and then the scapula before the elbow and then the hands. In this subsection, we explore the palm stretch and polishing glass actions as a way to open up the scapula for looser and more economical movement before moving onto the Kua. 

**Figure 2:**
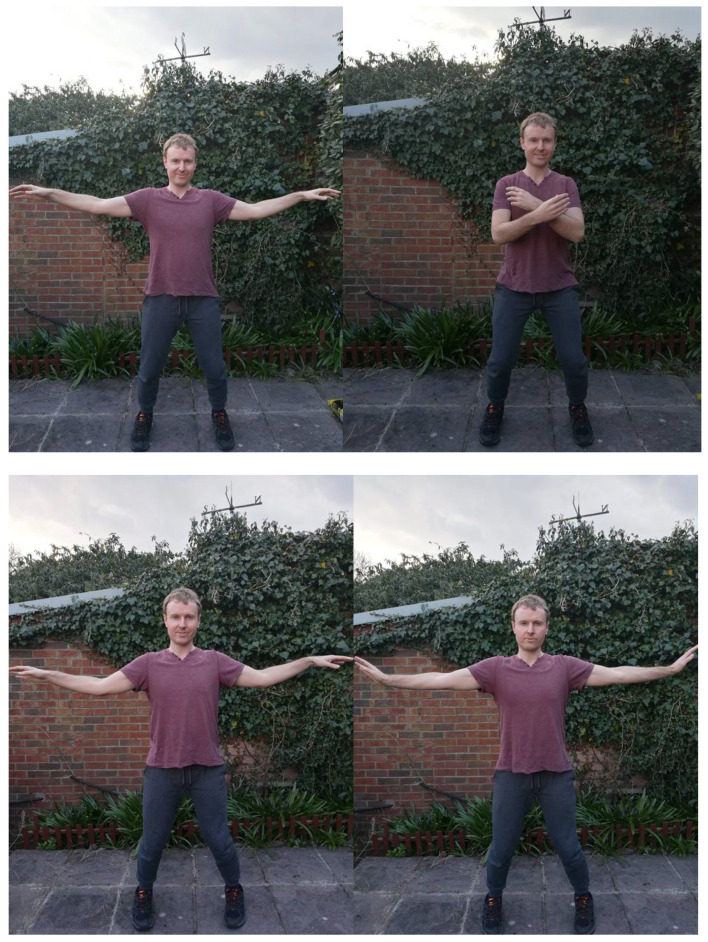
Shoulder Loosening Exercises and the Palm Stretch.

In Taijiquan and Chinese medical terminology, the center of the palm is known as the Lao Gong, which is believed to be a vital energy point [49]. The palm stretch develops a connection to the Lao Gong, but this is first performed by stretching the fingers of both hands in a lateral fashion as in a crucifix. The shoulders and elbows are then relaxed by the mind until they slowly drop, which enables the palms to rise up, with the fingers facing the ceiling. The practitioner then presses the palms out to an imaginary wall by pushing from the scapula. David stresses the importance of relaxing the upper or outer parts of the arm and shoulders, and moving from the important armpit region (as Taijiquan techniques require space around this region). George shows the start and finish points of this exercise in the lower row of images in Figure 2

Very often in class, the palm stretch is followed by the polishing glass exercise. Here, students hold out their arms in front of their bodies and draw them back several inches by pulling back the scapula and then opening them out, only to push them forwards again. “Like you’re polishing glass”, David reminds us regarding this motion, which stays at the same height all the way through the cycle. He regularly explains the importance of scapula mobility in Taijiquan, and this is exemplified in repeated motions in the short form. 

When practicing Taijiquan, the body is required to relax from head to toe and from the inside to the outside. No part of the practitioner’s body should be stiff. In this way, the practitioner can concentrate her/his thoughts, breathe peacefully, and move continuously. Loosening exercises as seen in the top row of images in Figure 2 are therefore used in David’s class to sink the scapula and release tension from the body through the articulations of the shoulder joint, the elbow joint and the wrist joint. During Taijiquan training, shoulder joint relaxation is the most important factor. Only when the scapula of the practitioner relaxes, can the person’s arms become soft and flexible. It is difficult for beginners who practice Taijiquan to loosen the scapula from the outset; only under the guidance of mind and after long-term exercise, can the shoulder joints slowly reach a relaxed state. Generally speaking, to relax the shoulder joint is to make the scapula sink. The shoulder joints of the practitioner must be level with both shoulders to relax and sink, not one high and one low, so that the elbow joint can relax and hang down. To relax the shoulder joint of the practitioner, the elbow joint must not be close to the ribs. Under the premise that the practitioner’s shoulder joints are relaxed, then the entire arm can be relaxed. Therefore, in the process of practicing Taijiquan, the first thing to do is to relax and sink the shoulder joints. Only in this way can we meet the Taijiquan practice requirements of “sinking shoulders, falling elbows and sitting wrists” (沉肩坠肘坐腕) [43] (pp. 162–163).

### 4.4. Opening the Kua through Kua Work

As the reader can see, there are many complex concepts for the student and teacher of Taijiquan to remember during class and home training. The aforementioned Kua is the turning point of the waist and legs. If the Kua is not loosened, the waist and legs cannot be flexibly coordinated. Of all the joint “Song” exercises, the Kua is the most difficult, as David notes from his numerous years of trying to loosen the waist and hips after a long period in Shotokan Karate in which he, like all his students, had “hardwired tension”. There are five main types of Kua loosening exercises in the School of Internal Arts. Regardless of the method, one must pay attention to the loosened waist and sinking of the Kua during each transition between techniques. Only in this way can the Kua loosen as the training time increases and the training experience deepens. David explained in one class:

*“The old adage of ‘you are what you eat’ is true. You are what you do. Or not what you do—how you do it. If you train with tension, you’ll become tense. If you train with anger, you’ll become angry. If you train relaxed, you’ll become relaxed”.*

The mention of the Kua as part of a rich theory on the subtle/energy body is another unusual aspect of the School of Internal Arts, although David recently reflected: “Ten years ago, no one was talking about the Kua, but if you go on YouTube now, plenty of people are mentioning the Kua”. This reflects a growing interest in the internal principles of Chinese martial arts, as in re-evaluations of popular “external” systems such as Wing Chun, which is now regarded as an internal system. Social media is playing a role in this revalidation and renaissance of styles, as seen in The Martial Man’s investigative videos shared by Andy, one of George’s classmates, who moved all the way from South Africa to train with the School of Internal Arts in the UK and Portugal (where he later resided). Andy devotes his life to self-cultivation and the development of the Taijquan-Neigong body through regular classes and theoretical study of the training principles through podcasts and books by the Academy and its esteemed peers.

**Figure 3:**
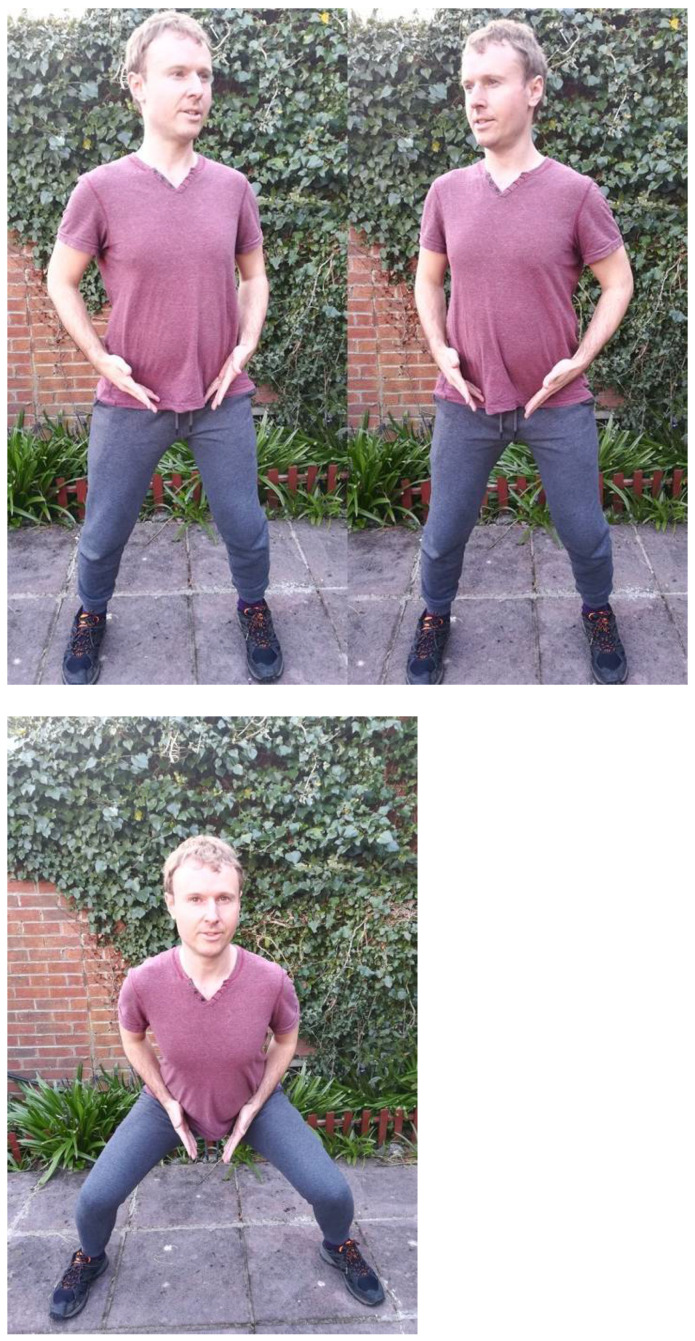
Kua Movements: Left, Right and Down.

Recently, in his own online class, David has been fond of a new phrase: “Principles, principles, principles—it’s all about the principles”, often adding utterances based on this need for bodily repetition, such as: “You can never do enough Kua work” in his calming tone, as students move through hundreds of repetitions of slight turns in their torsos. In fact, after two years in his class, George has gone through what must be thousands of repetitions of specific Kua movements. In one of the most common routines, students “pin the knees” as their teacher instructs them, and try to isolate the slight 30-degree angle from the invisible point of the Kua. With the command “place your fingers against the Kua”, they are sometimes asked to gently press their fingertips against the superficial area on the outer body, which equates to the pubic triangle in between the hips. Students like George slowly feel their fingers pressing into the soft tissue and glands as the Kua drives the movement, which might take several seconds to move to the left, back to the center and then to the right. Due to the intimate nature of this region, David never touches students there, although he might ask their consent to adjust other aspects of the posture from the upper torso, such as when they are leaning backwards or too far forwards. George has demonstrated this personal touch on one’s own Kua region in Figure 3, which demonstrates moving to the left and the right as well as sitting deep into the Kua in a Ma Bo stance.

Sometimes in class, students do perform contemporary fitness exercises such as crunches, the plank and brief press-ups for general fitness and to get into “the zone” for training. However, in the main part of the sessions, these seemingly Western exercises are absent, as the tuition focuses on generic Daoist physical cultural and medicinal concepts. “Kua squats” turns the familiar “Western” exercise of (bodyweight) squats into a more Chinese Daoist form of movement. As with some of the shoulder loosening exercises described above (as in the upper row of Figure 2), we move up and down according to three different heights—transitioning from a lower extremity to a middle point and then an upper position. This continues with the trend of pulling up the head from the occipital bone and lowering the tailbone with one’s intent (Yi). So, when one moves down from the Kua (as opposed to the knees), the head is still pulled up in order to continue with an erect spinal column. 

This Kua is likened to a gate that needs to be constantly opened and closed during practice. This is especially evident when running through the form sequence, when David calls us to “close the right Kua” or “close the left Kua”. The main action is therefore the closing, while the opening is a more responsive, passive movement. The Kua is also understood as a seat for standing and bearing one’s weight during the form sequence. “Sit into the Kua” is another common instruction as students shift the weight (or mass in Taijiquan terms) and try to sustain it, though not in our knees (as David warns us against doing), but within the Kua. David likens this to “sitting on a bar stool”, wherein the leg muscles do not tense, as the body is supported by the stool. This concept is taken into the fundamental exercise of standing examined next, which takes the sinking of the mass (Song) into the feet and ground. 

### 4.5. Compressing the Feet: Ba Men and Standing

**Figure 4:**
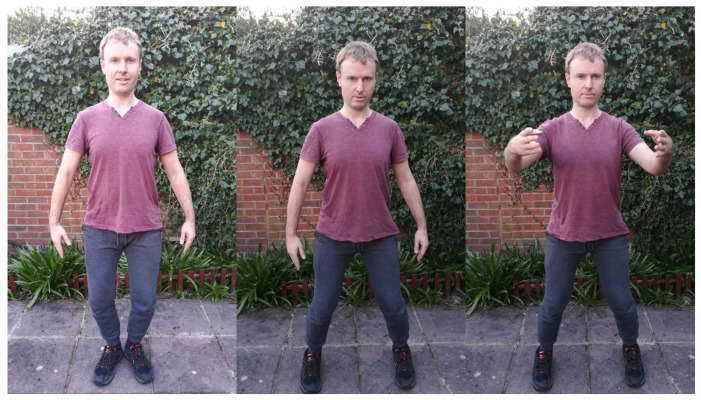

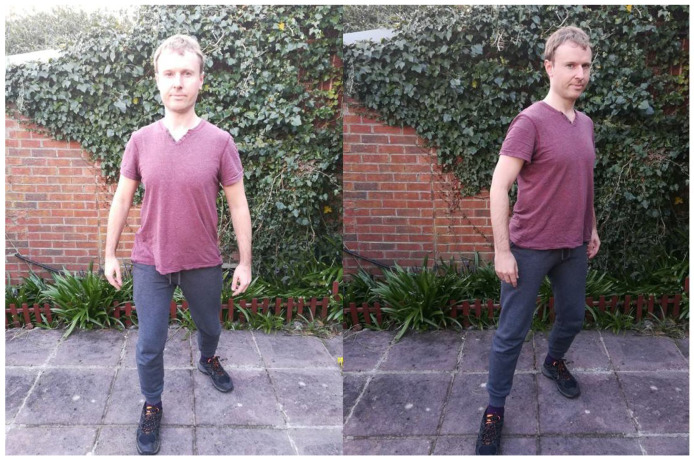
Three Training Stances of Opening Stance, Neutral Stance and Zhan Zhuang plus Ba Men forwards and backwards.

Standing in specific postures is the staple exercise of many Chinese “internal” martial arts as practiced according to the Yijinjing principles and theory. In David’s class, students often hold specific stances for minutes, and this is especially the case for the opening stance seen at the top left side of Figure 4 (heels together, with the feet opened like a ballet dancer), which is regarded as a rare yet fundamental position in the School’s particular lineage, which has a South East Asian influence. As with all the other exercises explained in the previous subsections, the head is held upright from the occipital bone and crown point, the chin is tucked in, the chest is soft and relaxed, the back straightened, and the sacrum expanded with space around the armpits. George has spent extensive periods of home training, holding this and other positions during his free evenings while listening to instrumental music. He often mixes the opening stance with the neutral stance (top middle picture in Figure 4), which involves standing around in a shoulder-width position, toes slightly turned inwards so that the second toe is central, sitting into the Kua with the arms by the side. Through one’s intent, students can (from the scapula) start to turn their hands so that their palms face behind them. After an extensive period—perhaps 20 or 30 min—the feet will begin to feel an immense amount of pressure. Within class training, George has joined his fellow students in slowly limping across to get a sip of water during a brief break between exercises. 

The agonizing training is particularly painful in the lower legs and soles of the feet, and this is particularly the case for the Wuji stance in the Neigong part of the 2.5 h class. This stance enables the students to close their eyes for Neigong, whereas in Taijiquan the eyes should always be kept open and alert. “If your calves are hurting, that’s a good sign”. These “achy calves” in training might be later accompanied by an “achy Kua” feeling after a hard evening’s training. One exercise that can be draining yet richly awarding at the same time is the Ba Men (or “Eight Gates”) exercise. Although there are eight complex movements with the whole body, we normally run through the first four before moving onto the intricate techniques such as the elbow and shoulder bump exercise, which has more obvious martial connotations. All movements are driven by the closing of one Kua and the sinking of the mass onto one foot, followed by the release of mass from that foot and the closing the opposite Kua. David sometimes works on this sequence at home for up to an hour on each side, although he advocated following one of his senior students Sean’s approach of drilling the basic footwork movements for ten minutes at a time on each side. “He’s found his root now; you can’t take that away from him!” marveled David, proud of his student’s accomplishments. To emphasize the Kua and lower limb movements, George demonstrates the footwork movements for Ba Men without the accompanying hand motions in the lower part of Figure 4.

Zhan Zhuang, seen at the upper right part of Figure 4, is a basic yet demanding exercise of Taijiquan, including “Song Gong” and “Zhuang Gong”. “Song Gong” aims to relax the whole body through the muscles, tendons and joints of the whole body. The main thing is to loosen the shoulders and elbows, loosen the waist, and then relax the whole body. Yang Chengfu also said, “Footwork should be divided into virtual and solid; ups and downs are like cat steps, if the weight is moved to the left of the body, the left is solid, and the right foot is virtual; when it is moved to the right of the body, the right is solid, while the left is empty” (虚实宜分清, 迈步如猫行, 左移则左实右虚, 右移则右实左虚) [43] (p. 36). 

In one evening class, students learned to walk in a Taijiquan fashion: continuing with the spinal elongation and being careful not to press on the heels as the weight was distributed through the padded soles of the feet. Pulling from the Kua, students transfer their weight from the back to the front legs at angles of around 45 degrees, moving from one side of the school hall to the next. During a quick water break, a critically-minded beginner student, Luke, remarked in a jovial fashion: “Go to a primary school to learn to walk!” to which the ever-enthusiastic Rich replied with a beaming smile: “Where better to learn?!” These men were re-learning to walk in that cat-like manner of alertness, crispness and control as advised by Yang Chengfu a century ago. As mature adults, they are undergoing a process of physical re-education decades after their formal physical education as school pupils—a process that aims to remove mental, emotional and physical tension built up through work, study and stress in everyday life. This discussion has therefore offered a description and an illustration of how adult British students of one Taijiquan and Neigong organization re-educate their bodies through a focused on relaxed, controlled movements in order to develop an idealized Taijiquan-Neigong body that attempts to “hang the flesh off the bones”.

## 5. Conclusions and Implications

All humans have different body shapes, sizes and abilities, with varying mobility and balance, and this is shaped by the diversity of body projects [50] available to us, from bodybuilding to jogging. Some adults in countries such as Britain are dedicating time and energy to develop a markedly different body (mind) through non-Western disciplines such as Yoga, Qigong and Taijiquan. In terms of the last two popular practices, this article has sought to address the research question: how, as adults, are Westerners learning to re-educate their varied, individual bodies through prolonged and intense Taijiquan and Neigong training? 

In order to explore this complex question, this article has drawn on linguistic data from an ongoing ethnographical and netnographical study of one branch of an international Taijiquan and Neigong association, the School of Internal Arts. This is a rather atypical Taijiquan association due to its emphasis on related Neigong theory and practice as connected to an interpretation of the core principles outlined in the early Yijinjing classic. The research design of ethnography and netnography (online ethnography) allows for the exploration of this re-education due to the prolonged and intense nature of the pedagogical strategy taken by this School in its regular classes, courses, online lectures and Zoom classes. From the two years’ fieldwork and online analysis, our initial finding was of the teacher and his students’ pursuit of a gradually integrated Taijiquan body (mind) as connected with important loosening exercises and other Neigong practices (meditation, Qigong, Daoyin and Neigong sets), i.e., what we call the Taijiquan-Neigong body: a specific bodily ideal that the members of the School of Internal Arts strive towards. For reasons of space and to focus on the key aspect of the pedagogy (exercises), this article has examined specific exercises in line with the development of specific parts of the body as understood by a Chinese Daoist medical paradigm. This subtle or energetic body is primarily concerned with the inner aspects of the body such as the scapula and armpits rather than the shoulder and arm muscles, and the Kua, sacrum and tailbone as opposed to the buttocks or quadriceps, along with the center of the feet—all of which combine to sink the mass for martial arts development. Through the cultivation of control and expansion of the joints, fascia and muscles, the Taijiquan-Neigong practitioner is expected to develop a “strong, powerful body” in David’s terms, which, as a by-product, is meant to be a healthy mind and body for daily living.

The second main finding is that, beyond a form of physical re-education, Taijiquan and Neigong enable the development of both the mind and the body, especially through the combined emphasis on the principles of Song and Ting, as well as integrated concepts around “hanging the flesh from the bones” and “sinking the mass”. Indeed, inspired by the philosophy of Yuasa [1,2] we highlighted the focus on mind-body integration through slow, meditative and repetitive movement can regarded as a form of self- (and, as Author [13] has argued, shared) cultivation, developing the human being in a rounded sense through cultivating habits and dispositions in terms of concentration, relaxation and emotional control. Following Spatz [33], we can see how humans can come to learn about themselves and the world through specific techniques repeated thousands of times in this intensive training, which is probably only suitable for adults willing to endure pain through immersion in their experience. The School of Internal Arts is an example of the voluntary physical re-education of adults following their earlier formal/compulsory physical education, lifelong habits, injuries, etc. Many of the members of the group come from backgrounds in Yoga, Qigong and the martial arts who are looking for something deeper, as the teacher David himself (a former Karateka) openly admitted to wanting when he found his own teacher and mentor, Malcolm. Through the Yijinjing principles (mainly “skeleton up, flesh down”) and concepts such as Song and Ting come the systematic removal of habitual tension developed over time while loosening the body to become springy and alert. This could have many positive implications for wellbeing in later life, which could be explored in future interdisciplinary research projects. 

Third, expanding popular physical fitness and beauty ideals, this ideal Taijiquan-Neigong body differs considerably from the conventional Western modes of physical education and its chiefly European sports, games and physical exercises. As [51] has indicated, while some children thrive under this games-based education, others learn a sense of helplessness (learned helplessness) that is hard to remedy within a semester or school year. In fact, the removal of physical, emotional and mental tension may take years, if not decades. Adults come to Taijiquan for many different reasons such as spiritual growth, the quest for relaxation after work and the continuation of careers as martial artists—themes currently being exposed in a series of interviews with members of the School of Internal Arts. In fact, Taijiquan and Neigong are primarily taught to adults as a form of physical re-education. Following the premise that education and learning can be lifelong ambitions, we can argue the case for alternative pedagogies for mature and older adults as being of particular interest to physical educationalists—be they teachers, researchers or policy makers. To reiterate: as education is lifelong, so is physical education. Yet re-education is much harder than education, as years of tension and tightness in adult body-minds require years or even decades of dedicated practice. This is something to be considered by physical educationalists in terms of policy, practice and pedagogy. 

It is important to note that as an ethnographic study of one association in Europe, this research project does have some limitations in terms of its specific focus on a particular school branch in Britain with a relatively unusual interpretation of the Yijinjing and Taijiquan. This article is therefore not intended as a generalized reading of all Taijiquan, but one specific interpretation of some of the fundamental solo body conditioning exercises of a hybrid lineage of the Yang family. It is also based on data taken from ethnographic (face-to-face) and netnographic (online) fieldwork, while the one-to-one interviews are still being planned during the COVID-19 pandemic. With these limitations of this ongoing project in mind, other studies investigating the philosophy and practices of different kinds of Taijiquan schools would help shed light on how people are learning to use their bodies in new and different ways through a re-education process. 

This is an early output from what is planned to be a five-year ethnographic study of the School of Internal Arts. The specific theory and practice of Neigong should also be explored in its own right. Future research might consider the role of core texts and elements of Chinese culture such as the Daodejing, Chinese astrology and medicine, which influence the interactions and practices of the group. For instance, some of the core members of the School have undertaken courses in Chinese medicine and acupuncture, and many of these students are now offering astrological services to their classmates, showing a Chinese cosmological view of the world, but demonstrated by non-ethnic Chinese people (as they are mainly white British and Europeans). We are planning a larger project to investigate the transmission of specific elements of Chinese culture outside of mainland China in countries such as Britain, where the dissemination of knowledge is often via online YouTube channels and podcasts as led by a new generation of reputed, mobile and charismatic instructors. 

Moreover, future research can consider broader approaches to applying Eastern philosophical principles in Western pedagogical contexts that move beyond classical ideas of the Yijinjing and Taijiquan in Britain. Continuing from Yuasa’s [1,2] overarching perspective on self-cultivation, [52] notes that, despite the great variety of Eastern models and cultures from Asia, there is a general interest in training the person in a holistic sense (all parts of the body aligned with the mind) and over the lifespan (rather than in a short, overly specialized sporting career). This has been raised by Lee [53] in his empirical research on children’s formal physical education, which noted the different approach taken in Western education such as focusing on the dominant hand when learning the overhand throw. Research on handedness, balance between the sides of the body and elements of the anatomy in Eastern martial arts would therefore be pertinent in comparison with those of the West (as in Taijiquan with its balanced approach to the body and Western (contemporary and historical) fencing, with its focus on the dominant side). This could accompany the exploration of philosophical ideals in other Eastern movement forms such as Yoga, Qigong and meditation which continue to flourish in Western contexts, albeit in often hybridized and commercialized formats [54]. 

## Data Availability

The data presented in this study are available on request from the corresponding author. The data are not publicly available due to the ethical agreement with the Cardiff Metropolitan University Social Sciences Ethics Panel to keep the data under George Jennings’ personal OneDrive account, which is not accessible to the wider public.
